# A Survey on Temperature-Aware Routing Protocols in Wireless Body Sensor Networks

**DOI:** 10.3390/s130809860

**Published:** 2013-08-02

**Authors:** Christian Henry Wijaya Oey, Sangman Moh

**Affiliations:** Department of Computer Engineering, Chosun University, 375 Seoseok-dong, Gwangju 501-759, Korea; E-Mail: chwijaya@gmail.com

**Keywords:** wireless body sensor network, routing protocol, temperature awareness, bioeffect, wearable computer

## Abstract

The rapid growth of the elderly population in the world and the rising cost of healthcare impose big issues for healthcare and medical monitoring. A Wireless Body Sensor Network (WBSN) is comprised of small sensor nodes attached inside, on or around a human body, the main purpose of which is to monitor the functions and surroundings of the human body. However, the heat generated by the node's circuitry and antenna could cause damage to the human tissue. Therefore, in designing a routing protocol for WBSNs, it is important to reduce the heat by incorporating temperature into the routing metric. The main contribution of this paper is to survey existing temperature-aware routing protocols that have been proposed for WBSNs. In this paper, we present a brief overview of WBSNs, review the existing routing protocols comparatively and discuss challenging open issues in the design of routing protocols.

## 1. Introduction

According to the Department of Economic and Social Affairs of the United Nations Secretariat, the elderly population (persons of age 60 years and over) in the world in 2010 was 759 million and projected to be 1,198 million in 2025, or 15% of world population [1]. Since the people belonging to this age group are susceptible to various health issues, they tend to require more frequent healthcare treatment. However, it is considered inconvenient and costly if they have to periodically do medical check-ups in healthcare facilities that are located far from their home, not to mention the fact that they are much less mobile than younger ones. Moreover, the cost of healthcare for the elderly is more expensive than that for other aged groups. Since most of healthcare expenditures are intended to serve elderly people, this situation could become a big challenge in the future if not taken seriously, given the limited resources.

Besides the healthcare cost issue, the traditional health monitoring system imposes the problem of inaccuracy. Usually, patients are monitored only at a certain point of time, and the next monitoring occurs after a considerable period of time. This would lead to incomprehensive health diagnosis. For example, a healthy patient might be diagnosed to have high blood pressure, whereas, in fact, that misdiagnosis is caused by exhaustion after he walks to the clinic. Therefore, a non-intrusive, ambulatory, continuous, yet economical health monitoring system needs to be developed to achieve a better and complete picture of health diagnosis and reduce the cost of healthcare.

Currently, small and intelligent sensor devices can be attached on or even implanted into the human body, thanks to the advancement of microelectronics and micro-electro-mechanical systems (MEMS). These battery-powered devices gather patients' vital signs and send it to medical workers, e.g., physicians and nurses, for further examination and analysis. There might be more than one sensor device attached to the body. The wired connections between devices for collecting data are not effective, troublesome or even impossible for daily use. As a solution, the sensors are equipped with wireless transceivers, so that they can communicate wirelessly to transmit the sensed data. This type of network is called Wireless Body Sensor Networks (WBSNs) or, sometimes, also called Body Sensor Networks (BSNs); hereafter WBSNs. Since WBSNs are actually a subset of Wireless Sensor Networks (WSNs), WBSNs inherit the typical challenges and issues of WSNs.

WBSNS are a wireless network optimized for low power devices and operation on, in or around the human body (but not limited to humans) to serve a variety of applications, including medical, consumer electronics/personal entertainment and others [2]. By this definition, WBSNs can be used in many application areas. However, current studies in WBSNs are mostly focused on medical applications, since this is the main purpose and reason for developing WBSNs.

**Figure 1.**
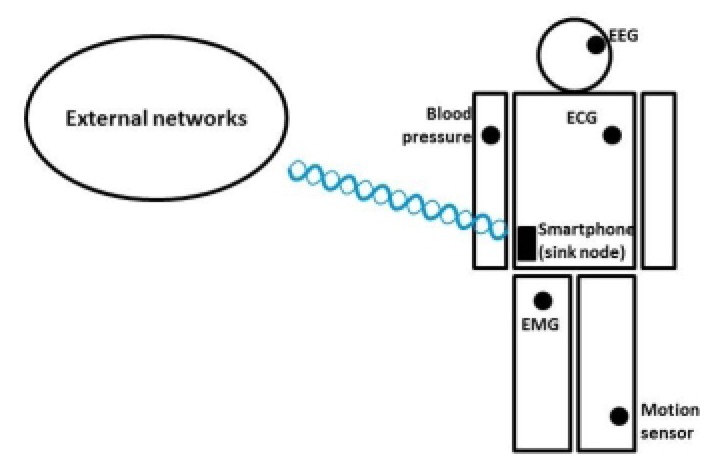
A general example of Wireless Body Sensor Networks (WBSNs).

A WBSN consists of one or more sensor devices positioned on, in or around the human body. The sensor devices sense and collect data from the human body and then transmit the data to a central device, called a sink, that can be in the form of a smartphone or PDA. After collecting all information, this sink then forwards the data to the medical workers through external networks. A general example of WBSNs is shown in Figure 1.

Despite the promising applications for healthcare systems, WBSNs also impose several challenges in network design and implementation. As the devices are attached to the human body, a careful design must be made to protect human tissue from heat caused by the radiation and device operation. Human safety becomes the most important factor in WBSNs. Another challenge is about energy efficiency. In several applications, devices are intended to operate for a long period of time. Therefore, it is not practical if the devices require frequent battery replacement, considering their location inside the human body.

Ullah *et al.* [3] provided a comprehensive survey on WBSNs. They surveyed WBSNs on PHY, MACand network layers and classified routing protocols based on their strategies: temperature-aware, cross layer and clustering. In this paper, however, we focus only on the temperature-aware routing protocols, because temperature rise directly affects human health and safety, as studied in [4,5]. The antenna radiation and node circuitry operation generate and release heat to their surroundings, which can be harmful to the human body if a safety threshold level is surpassed. The Federal Communications Commission (FCC) standard states that the upper limit of specific absorption rate (SAR) for near-field exposures is 1.6 W/kg for any tissue averaged over one gram, whereas the International Commission on Non-Ionizing Radiation Protection (ICNIRP) defines the upper limit of 2.0 W/kg for 10 g of tissue. The study in [4] translates these SAR values into temperature rise, and the maximum values of possible temperature increase in the human head and brain are 0.31 °C and 0.13 °C for the FCC standard and 0.60 °C and 0.25 °C for the ICNIRP standard, respectively. The study in [5] also indicates that a temperature rise of 0.1 °C is high enough to trigger intense human body thermoregulatory responses. That is, the temperature rise is the most important factor in designing a routing protocol for WBSNs, and how a routing protocol operates has a direct effect on the node activities, which, in turn, affect the temperature rise. To the best of our knowledge, no survey focusing on the temperature-aware routing protocols in WBSNs has been conducted.

The rest of the paper is organized as follows. Section 2 presents the challenging open issues in WBSNs. In Section 3, the explanation of each routing protocol surveyed in this paper is provided. The comparative discussion of the routing protocols is presented in Section 4. Finally, this paper is concluded in Section 5.

## 2. Challenging Open Issues

Generally, a routing protocol can be defined as a set of rules to successfully deliver data from a source to a destination node. Designing a routing protocol for a specific environment is not trivial work, as it is influenced by many challenging issues and factors that must be overcome to achieve a particular design objective. As mentioned earlier, WBSNs inherit the typical challenges and issues of WSNs. However, WBSNs have more specific requirements, due to their placement in the human body. They must put human safety as the top priority in the design over other requirements, such as packet delay or packet drop rate. In this section, we discuss the challenging open issues that need to be considered when designing a routing protocol in WBSNs, with emphasis on bioeffects as the determining factor that distinguishes itself from WSNs.

### 2.1. Bioeffects

The unique characteristic of WBSNs is that the nodes are located inside, on or around the human body. The node operation will produce heat and cause temperature rise in its vicinity. When the node's power consumption is very low or the node is not actively sending data, it might not generate significant heat. However, when the node is operating continuously, transmitting and receiving the data in a considerable period of time, the heat generated by the node cannot be neglected. This concern becomes even bigger when dealing with in vivo sensor nodes (i.e., implanted inside the human body). The human body has a thermoregulatory mechanism to balance the heat around the body. However, when the heat received rate is larger than the thermoregulatory mechanism rate, the temperature will rise and, in turn, damage the human tissue.

Lazzi [6] conducted a study about the thermal effects of bioimplants. There are two main sources of temperature rise when sensor nodes are implemented in the human body. They are the power dissipated by the implanted sensor nodes and the electromagnetic fields induced in the human body. The power dissipation itself can be divided into three sources: the power dissipated by the implanted microchip, by the implanted telemetry coil and by the stimulating electrodes. When the node operates, it will consume energy, and there is some energy portion that is dissipated. The dissipated energy will be converted into heat and increase the temperature of its vicinity. The longer the node operates and transmits/receives data, the more energy will be dissipated and turned into heat.

The sensor nodes implanted inside the human body can be safely assumed to be using a wireless system to transmit and receive the data. The human tissue will absorb the radiation energy and convert it into heat, which in turn, will increase the temperature. The well known parameter used by most international standards regarding the electromagnetic safety toward the human body is specific absorption rate (SAR). It can be defined as a measure of the rate at which energy is absorbed by the body when exposed to a radio frequency electromagnetic field, expressed in W/kg. By looking at its unit, we can also say that SAR shows the power dissipated per unit mass of tissue. The value of SAR is determined by these four factors: tissue density, conductivity and electric field amplitude at a point of location in human tissue. Therefore, based on where the SAR value is calculated, the value can be different depending on those factors. However, the IEEEstandard recommends the value of 1.6 W/kg averaged over one gram of tissue as the acceptable value of SAR. This value is also adopted by FCC (Federal Communications Commission) to regulate the SAR level of mobile phones sold in the United States. The readers are encouraged to read [6] to get a more detailed explanation about the thermal effects of bioimplants.

The effects of these factors actually can be reduced by a good hardware design. A node and its antenna can be engineered and designed to consume as low energy as possible, which, therefore, reduce the temperature rise. In addition, a properly designed routing protocol used in the network also plays an important role in reducing the bioeffects, and this is what the routing protocols presented in this paper are trying to accomplish.

### 2.2. Other Open Issues

In this subsection, the other open issues related to the design of routing protocols in WBSNs are presented.

#### 2.2.1. Network Topology

There are two main types of network topology, depending on how many hops the data traverses from the source node to the sink node: single-hop and multi-hop topology. A single-hop topology means that every sensor node is directly connected to a sink node, while in the multi-hop topology, the data transmitted from the sensor nodes to the sink node will traverse through one or more intermediate nodes before reaching the sink node. In case of single-hop, no routing protocol is needed, since all sensor nodes are one hop away from the sink node. However, due to the lossy nature of the human body, it is not always possible to have a direct communication between the source node and the sink node. It is more likely that the data will go through intermediate nodes before reaching the sink node. Moreover, Natarajan *et al.* [7] conducted an experiment to investigate the reliability between the two topologies. The packet delivery ratio parameter is used to measure reliability. It turned out that the multi-hop topology is more reliable than single-hop topology. Based on the reasons above, we can conclude that for WBSNs, multi-hop topology is the best choice.

#### 2.2.2. Packet Delivery Delay

The packet delivery delay in WBSNs also plays an important role. As the typical applications of WBSNs are in the medical area, the packet usually must be delivered from the source node to the destination node within a certain period of time or deadline, otherwise it is useless. How to reduce the delay as low as possible while maintaining the temperature level becomes the challenge in designing a routing protocol in WBSNs. The routing protocols in [8–11] try to mitigate this issue.

#### 2.2.3. Energy Consumption

WBSNs designed for human use must be noninvasive and ambulatory. Thus, it must have fewer and smaller nodes, which also implies a smaller energy capacity. Because of this, one has to consider the trade off between the energy capacity and energy consumed by the processing and communication operations in order to use energy efficiently. The routing protocols in [12–15] try to mitigate this issue.

#### 2.2.4. Path Loss

In WBSNs, sensor nodes might be placed on the body, e.g., chest, back, wrist or inside the body, e.g., pacemaker for regulating heart beating. The wireless transmission between these nodes must propagate through the human body, and compared to free space medium, which has a path loss exponent of two, the human body is an extremely lossy environment. The path loss exponent in the human body ranges from four to seven [16], a value relatively much higher than the condition in free space. The signal power will be severely degraded; thus, this factor must also be considered in designing a routing protocol for WBSNs.

#### 2.2.5. Reliability

Reliability in the data delivery in a network can be measured by Packet Delivery Ratio (PDR) and Bit Error Rate (BER). PDR represents the ratio of the number of packets received by the receiver to the number of packets generated by the sender, while BER represents the ratio of the number of error bits to the number of bits generated by the sender. Reliability is very important in medical applications. An error in data transmission from the sensor node to the sink node could be fatal and could lead to mistreatment. Thus, the routing protocol design must consider how to deliver the data as reliably as possible.

#### 2.2.6. Node Heterogeneity

In WBSNs, sensor nodes are most likely heterogeneous. Each sensor node with its specific application has different requirements and constraints. Each node might have a different size and different capacities in terms of computation, communication and energy capacity. They also might have different Quality of Service (QoS) requirements, depending on what application they are intended for. One application might require a higher data rate, while another might be tolerant to a long delay.

#### 2.2.7. Data Aggregation

Because energy consumption for communication is much higher than that for computation [17], data aggregation might be considered as a way to save energy. Data received from different sources is combined and fused before being transmitted to the next hop node. Whether this technique is used or not, energy consumption must be considered, as there is a trade off between energy consumption and network load. When data aggregation is not used, this means that the packet size sent to the next hop node will be larger.

#### 2.2.8. Quality of Services

In every application, Quality of Services, or QoS, must be carefully considered. Each application has its own requirements: maximum delay, data rate, packet loss, bit error rate, etc. When these requirements are not met, there might be issues for the applications. For example, an electrocardiogram used for monitoring heart rate in the middle of surgery must provide a real-time measurement of the heart rate of the patient. If it exceeds the acceptable delay or latency value, then it becomes useless. The routing protocols in [18–20] try to improve the QoS.

## 3. Temperature-Aware Routing Protocols

**Figure 2.**
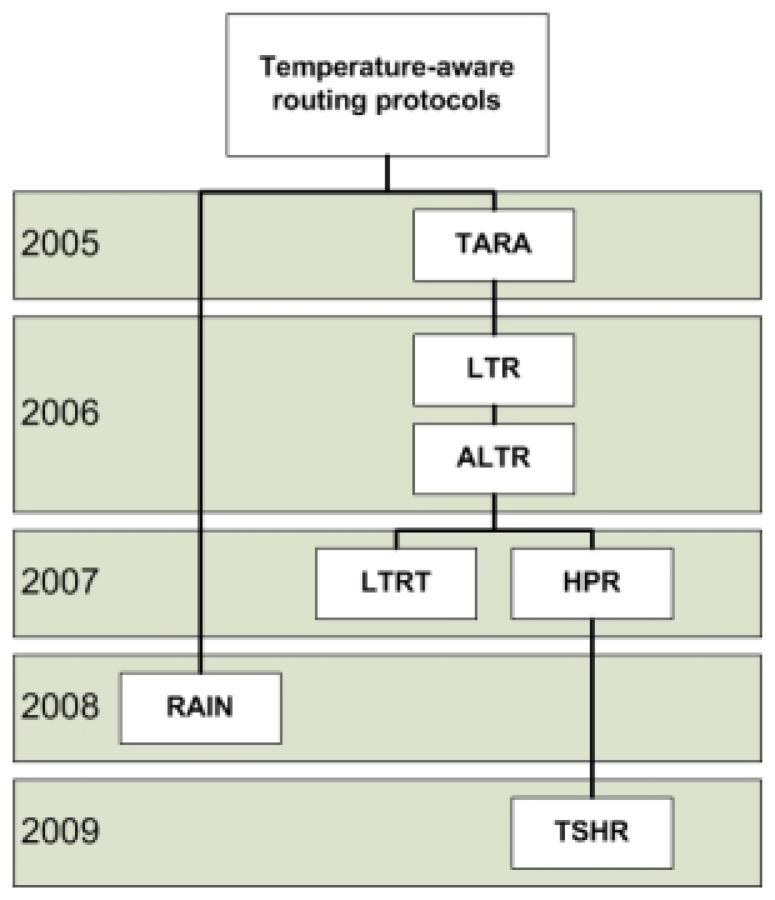
The taxonomy of temperature-aware routing protocols in WBSNs.

The routing protocols to be presented here are those that take node temperature as a metric in the routing path decision, and thus, they are called temperature-aware routing protocols. The purpose of the protocols is to keep the node temperature below the temperature safety level and to slow down the temperature rise rate, so that it does not harm the human body. The application of the routing protocols presented here is for in vivo sensor nodes, and the taxonomy of temperature-aware routing protocols in WBSNs is shown in Figure 2.

### 3.1. TARA (Thermal-Aware Routing Algorithm)

TARA, or the Thermal-Aware Routing Algorithm [21], is known as the first protocol that introduced temperature as a routing protocol metric. As explained in the previous section, TARA also considers two sources as the major sources of heat: antenna radiation and power dissipation of node circuitry. However, since nodes are small in size and expected to be as simple as possible, it is assumed that there is no temperature sensor inside the node to measure the temperature. Therefore, the temperature is measured by observing sensor activities, from antenna radiation and power dissipation of the node circuitry.

The general operation of this protocol is as follows. In the setup phase, each node exchanges neighborhood information, creates its own neighbor list and collects the number of hop information, so that every node knows how to reach the sink node. Next, in the data forwarding phase, the nodes having data to send will forward the packet to the next hop, until it reaches the sink node. A node whose temperature exceeds a predefined threshold value will be marked as a hotspot node, and any packet having a hotspot node as its destination node will be buffered until the estimated temperature drops. If the buffered packet period exceeds the timeout period, it will be dropped.

**Figure 3.**
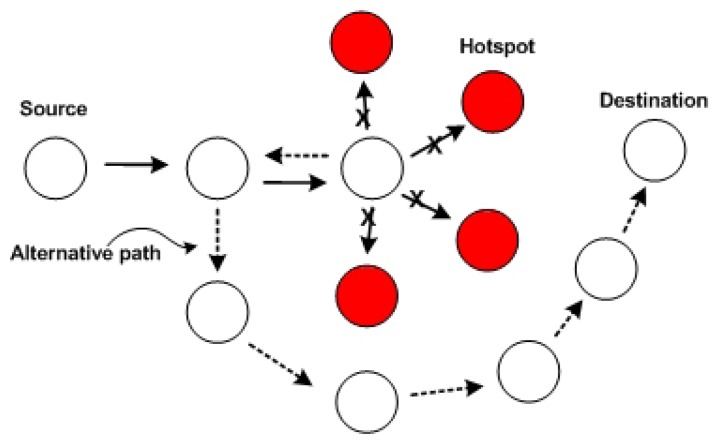
An example of Thermal-Aware Routing Algorithm (TARA).

When the hotspot node is an intermediate node, the packet will be routed through a different path and, thus, avoid the hotspot area. This strategy is called withdrawal strategy, which operates as follows. If the next hop node is a hotspot node, the node will check into the forwarding set of this route whether there is any other available next hop node to send the packet to. Should there be no more available next hop nodes, the packet will be forwarded back to the previous node. This previous node will try to forward the packet using an alternative path or might again forward it back to its previous node. The information about hotspot nodes is carried by the packet when the withdrawal strategy is used. A sample of how TARA works is shown in Figure 3.

Basically, TARA tries to avoid the hotspot area by observing the neighboring nodes' temperature and detour the packet using the withdrawal strategy. This strategy causes high delay and low network lifetime, since the packet will be roaming around the network for some considerable amount of time. However, this withdrawal strategy, which avoids the hotspot nodes, will balance the load in the network, since hotspot nodes are considered as high load nodes, i.e., nodes that already served a lot of packets.

### 3.2. LTR (Least Temperature Routing)

**Figure 4.**
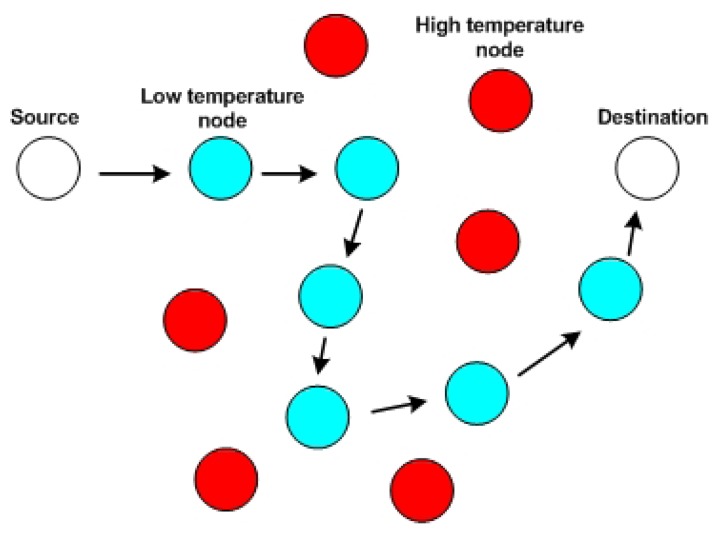
An example of Least Temperature Routing (LTR).

LTR, or the Least Temperature Routing protocol [22], is developed based on the TARA protocol. The setup phase is similar to that of TARA: every node communicates with its neighbors and gathers information about their temperature by observing their activities. The improvement lies in how the packet is forwarded in the network. Unlike TARA, which buffers the packet if the destination node is a neighboring node, LTR forwards the packet directly to the destination node. Additionally, as its name implies, in LTR, each node tries to forward the packet to the “coolest” neighbor since the beginning of transmission from the sender node. There is also a packet-discarding mechanism, in which a parameter, named MAX HOPS, is defined, and if the received packet's hop count exceeds this value, the packet will be dropped. The purpose of this mechanism is to prevent a packet from going around the network too far. Another mechanism to reduce unnecessary hops and loops is also presented. If the “coolest” neighbor has been recently visited, the packet will be forwarded to the “second coolest” neighbor. To support this mechanism, a list of recently visited nodes within some time window is included in the packet. Figure 4 shows a sample of how the LTR protocol works in the network.

### 3.3. ALTR (Adaptive Least Temperature Routing)

**Figure 5.**
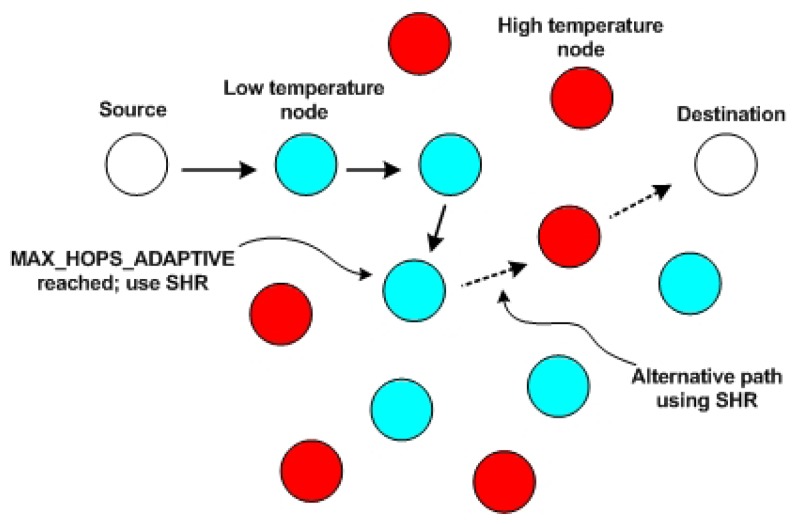
An example of Adaptive Least Temperature Routing (ALTR).

ALTR, or Adaptive Least Temperature Routing [22], is the adaptive form of LTR. A new parameter, named MAX HOPS ADAPTIVE, is presented by this protocol. Each time a packet is received by a node, its hop count value is examined. If the value is lower than MAX HOPS ADAPTIVE, then the packet is routed in the same way as LTR. However, as shown in Figure 5, if the value is greater than MAX HOPS ADAPTIVE, instead of being dropped, the packet is routed using the shortest hop algorithm. ALTR also introduced a mechanism called “proactive delay” to reduce the temperature rise at the cost of the packet delivery delay. If a node receives a packet and has no more than two outgoing neighbors, such that its coolest neighbor has a relatively high temperature, the node delays the packet by one time unit before forwarding it to the coolest neighbor.

### 3.4. LTRT (Least Total-Route Temperature) Routing

This protocol, named LTRT, or Least Total-Route Temperature [23], is basically a hybrid between the LTR protocol and shortest hop routing algorithm. This protocol uses temperature as its metric, and then, using the shortest hop algorithm, it calculates the route with the lowest temperature metric.

**Figure 6.**
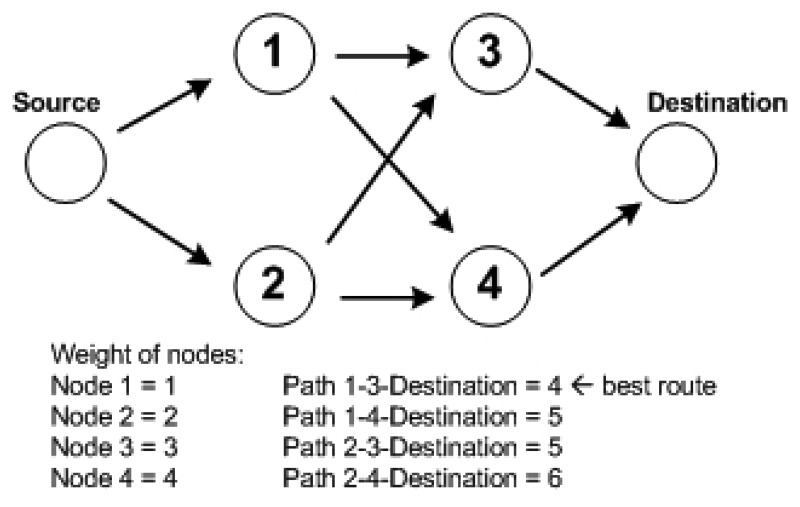
An example of Least Total-Route Temperature (LTRT).

The algorithm of this protocol is briefly described as follows. First, every nodes collects the temperature information from its neighboring nodes and, then, builds all possible routes to the destination. Then, it assigns temperature as a weight to each intermediate node and builds a weight graph. On this graph, it applies the Djikstra's algorithm to solve the single source shortest path (SSSP) problem of the weight graph. The result is a path that has the lowest temperature metric. A sample of this graph is shown in Figure 6. That being said, since the beginning, LTRT tries to look at the end-to-end connection perspective, instead of the connection between two directly connected nodes only. It tries to select the least temperature route from every possible route.

### 3.5. HPR (Hotspot Preventing Routing)

The Hotspot Preventing Routing, or HPR, algorithm [24] is an improvement of LTR and ALTR. In LTR and ALTR, reducing the network average temperature does not necessarily mean preventing any node from having a very high temperature. However, HPR not only prevents the formation of hotspot nodes in the network, but also prevents the packets from taking suboptimal paths and, thus, reducing the average network delay. This protocol achieves both objectives of preventing hotspots and reducing delay by means of the shortest hop algorithm and a threshold value.

There are two phases in HPR: setup phase and routing phase. In the setup phase, nodes exchange information about the shortest path and initial temperatures and build a routing table based on that information. In the routing phase, first, the nodes use the shortest hop algorithm as long as no hotspots appear in the path. A hotspot is dynamically determined using a threshold value that is derived from both the average temperature of neighboring nodes and the nodes' own temperature. If the next hop node's temperature exceeds the summation of temperature of the sender node and threshold, then the packet will be forwarded to the “coolest” neighbor. Similar to LTR, HPR uses the MAX HOPS parameter, which drops packets that exceed this threshold value, and a list of recently visited nodes to prevent any routing loops.

This protocol differs from its predecessors on how it defines when a node is marked as a hotspot. The previous protocols use a predefined temperature threshold value, and if a node exceeds this value, it is marked as a hotspot. However in HPR, the threshold value is dynamically calculated by each node considering the neighbors' temperature and its own.

### 3.6. TSHR (Thermal-Aware Shortest Hop Routing)

TSHR, or Thermal-aware Shortest Hop Routing [25], is an improvement of HPR. It is almost similar to HPR in that it has two phases—a setup and routing phase—and makes use of a threshold. The difference is that in TSHR, there are two kinds of thresholds introduced: a fixed threshold and dynamic threshold. A fixed threshold is a threshold value that is applied for all nodes, while a dynamic threshold is a threshold value that is specific to each node and set based on the temperature of the node and its neighbors. The dynamic threshold is used to mark a node as a hotspot. If the next hop node temperature exceeds this dynamic threshold, the sending node will look for another coolest node, which has not been visited by the packet. On the other hand, the fixed threshold is used every time a node is going to transmit a packet. It compares the next hop node temperature to the fixed threshold, and if it exceeds the threshold, the packet will be buffered until the next hop node temperature is below the threshold. The fixed threshold implies the maximum node temperature allowed in the network. Another difference is that no packets are dropped in TSHR. If the hop counts exceed the maximum hop threshold, then the packet is forwarded using the shortest hop algorithm.

### 3.7. RAIN (Routing Algorithm for Network of Homogeneous and ID-less Biomedical Sensor Nodes)

RAIN [26] stands for Routing Algorithm for Network of Homogeneous and ID-less Biomedical Sensor Nodes. The main difference between RAIN and the above routing protocols is that in RAIN, the nodes are ID-less. It is argued here that the overhead of ID maintenance is very high, and in sensor networks, how to get the sensed data from the network is more important than knowing the node ID itself. That is why the approach of using and ID-less network is proposed.

The word ID-less here does not mean it really does not use node an ID at all. It uses, instead of a static ID, a temporary ID. This ID is generated during the setup phase of protocol operation. Each node has a random number generator that generates a random number between one and (2^16^−1), which will be its node ID during operational lifetime. Since this protocol does not maintain global addressing, it relies on local addressing and coordination. Each node needs to know only its neighbor's ID; thus, there might be two identical node IDs in two different localities in the network. Nonetheless, this does not affect the network performance.

In this protocol, a mechanism to prevent a node from sending a duplicate packet is implemented. The algorithm will check whether or not the received packet ID is already in the queue. If it is, then the packet will be dropped. Hop count check is also implemented to prevent packets from going around the network uselessly. This protocol deals with node temperature by using a probability to send a packet to the next hop nodes. Each next hop node is assigned a probability value that is inversely proportional with the estimated temperature of the nodes. This way, the “coolest” neighbor node is likely to be chosen as the next hop node.

Another main mechanism implemented in RAIN is Status Update, a mechanism implemented to mitigate the “Energy Hole” problem in the network. The “Energy Hole” problem occurs when the nodes around the sink become depleted of energy very fast, due to receiving and forwarding packets coming from the network. To mitigate this, the sink node will broadcast a status update to its neighbors, which contains the packet ID of the received packets by the sink node. This will prevent neighbor nodes from forwarding multiple copies of the same packet to the sink node, thus saving a lot of energy.

## 4. Comparative Discussion

The ultimate objective of the temperature-aware routing protocols is to reduce the temperature rise caused by the nodes' operation. All protocols here are intended for in vivo sensor nodes, that is, the nodes implanted inside the human body. In this section, we first discuss the major features of each protocol, including its advantages and disadvantages, and then, the performance of the routing protocols are qualitatively compared. A discussion of how a routing protocol in WBSNs should be designed is also presented.

### 4.1. Major Features

In this subsection, the temperature-aware routing protocols are reviewed in terms of major features and, then, compared with each other qualitatively. The comparison results are summarized in Table 1.

TARA is the first routing protocol that introduced temperature as a routing metric. The first advantage of TARA compared to the SHR (shortest hop routing) protocol is that it achieves a better performance in terms of reducing the maximum and average temperature rise. The SHR protocol only cares about how to send the packet to the destination node using as short a route as possible without considering any other factors. While it achieves a low packet delivery delay, it suffers from high temperature rise, since the same nodes will always be used for a particular route. On the other hand, the TARA protocol is concerned with the temperature of the nodes and uses the withdrawal strategy; it will avoid hotspot nodes by sending back the packet to the previous node and tries another available route. This mechanism will reduce the average temperature rise of the network and limit the maximum temperature to the predefined threshold value. Nevertheless, as the trade-off, the withdrawal strategy introduces high delay, because the packet will be detoured arbitrarily using another route as long as there is an available next hop node.

The second advantage of TARA is reducing the traffc congestion in the network by means of load balancing. TARA calculates the temperature of neighboring nodes by observing the nodes' activities, that is, how many packets are transmitted and received by the nodes. It means that the more the number of packets that are transmitted and received by a node, its temperature will rise. Therefore, while TARA tries to distribute the temperature in the network, at the same time, the load of the network is also distributed, thus resulting in less traffic congestion.

The packet delivery delay plays an important role in routing protocols in WBSNs. The drawback of TARA, which introduces delay, due to the withdrawal strategy, is trying to be solved by the LTR protocol. Unlike TARA, if the next hop node is the destination node, the packet is forwarded immediately, with no buffering mechanism. There is also no withdrawal strategy in LTR, which means there is no packet sent back to the previous node, and a list of recently visited nodes is also maintained to prevent routing loops. Moreover, from the beginning, LTR tries to send the packet via the coolest neighbor. However, there is a packet discard mechanism to prevent a packet roaming around too long in the network.

The ALTR protocol is an adaptive form of LTR. The adaptive form, here, means that the general mechanism is the same as LTR, but a different threshold, called MAX HOPS ADAPTIVE, is introduced. When the hop count of the packet received by a node exceeds this threshold, the packet is forwarded to the destination node using the shortest hop algorithm. By means of these mechanisms, LTR and ALTR achieves a better performance than TARA in terms of packet delivery delay and average temperature rise in the network. In addition, while LTR still introduces packet drop, due to the packet discarding mechanism, ALTR achieves a comparable performance with SHR in terms of the percentage of packet drop during the simulation.

Another disadvantage of LTR and ALTR is that there is no guarantee that during the transmission from sender to destination, the packet will always be transmitted toward the direction of the destination node. A packet may be sent to a wrong direction away from the destination node, because LTR and ALTR only are concerned about the nodes' temperature. Moreover, in ALTR, the packets might be transmitted through the hotspots after exceeding MAX HOPS ADAPTIVE, which will result in temperature rise.

These disadvantages are trying to be mitigated by the next protocol, LTRT. Looking at the end-to-end connection perspective, LTRT transmits the packet through the path or route with the lowest temperature level. Therefore, the packet will be always transmitted toward the destination node, not sending the packet back to the previous node. Using this algorithm, LTRT achieves a lower average temperature rise and lower packet delay compared to LTR and ALTR.

Nevertheless, the average temperature rise metric that is used by LTR, ALTR and LTRT is not appropriate for WBSNs applications. The reason is that the average temperature rise does not reflect the individual node's temperature. The average temperature level may appear to be low, while, actually, there are nodes that already exceed the safe temperature level. These nodes that are already above the safe temperature level are hazardous for human health. Therefore, the metric maximum temperature rise, which is used by TARA, is more appropriate to be used in WBSNs, because it shows the maximum temperature rise that exists in the network.

The HPR protocol provides a better approach in dealing with temperature rise in the network. It considers the maximum temperature rise as the metric, not average temperature rise, and also, it uses a dynamic threshold based on the node's own temperature and its neighbors. HPR achieves a better performance compared to TARA and SHR in terms of maximum temperature rise, average delay and packet drop, especially in a high packet arrival rate environment. Unlike TARA, which tries to withdraw the packet back to the previous node, HPR tries to bypass the hotspot nodes by sending the packet to the coolest next hop node.

The TSHR protocol made some modifications on the HPR protocol. Besides the dynamic threshold that is used by HPR, TSHR added one more temperature threshold, called a fixed threshold, which is applied to every node before it transmits the packet. This threshold defines the maximum temperature allowed for a node to participate in the routing process. Different from HPR, in TSHR, there is no packet discarding mechanism. If the next hop node is a hotspot, the packet will be buffered, until the temperature drops below threshold. While this provides a low packet drop rate, it suffers from higher network delay compared to HPR.

As for the last protocol, the RAIN protocol offers a unique characteristic. In contrast with the previous protocols, RAIN does not use global ID for each node; it uses the local ID instead, which is generated randomly. This approach is developed to deal with the possibility of and ID-less network deployment in the future. This is a clear advantage of RAIN compared to the other protocols.

### 4.2. Performance

The performance of the above mentioned protocols were evaluated and compared in terms of maximum temperature rise, average packet delivery delay and packet drop rate in Figure 1, Figure 2 and Figure 3 of the work [25], respectively. The shortest hop routing (SHR) protocol was evaluated as the representative of non-temperature-aware routing protocols, and the four temperature-aware protocols of TARA, LTR, HPR and TSHR were evaluated quantitatively.

In terms of maximum temperature rise, the TSHR protocol outperforms all other protocols, while the SHR protocol is the worst. As for the packet delivery delay, SHR is the best and HPR is the second best. The TARA protocol performs the worst, because, in TARA, every time a node encounters a hotspot, it will send the packet back to its previous sender and try to find another path. This mechanism increases the packet delivery delay. The TARA protocol, again, performs the worst with regards to the packet drop rate, as opposed to SHR and TSHR, in the first and second place. A longer path in TARA causes the higher packet drop rate, whereas, in SHR and TSHR, the shorter path tends to reduce the packet drop rate. For more information, interested readers may refer to the performance evaluation results in [25].

The performance of the RAIN protocol is compared with that of a flooding algorithm, called C-FLOOD in [26]. It is reported that RAIN outperforms C-FLOOD in terms of maximum temperature rise, energy consumption, packet delivery ratio and average packet delivery delay.

### 4.3. Discussion

From the explanation above, we can see that all the routing protocols presented here have the same method to estimate a node's temperature. They calculate the temperature by observing the node's activities: how many packets are transmitted and received by the nodes. They also use a distributed algorithm in the sense that each node has the capability to decide the route; it does not depend on one node to decide the route.

However, after observing the advantages and disadvantages of each routing protocol, we can find some room for improvement in routing protocols for WBSNs in the future. Table 1 shows that all the protocols use a proactive or table-driven routing approach, which means that each node maintains a routing table to be used for the routing decision. However, the reactive or on-demand approach is also worth considering to be implemented in the routing protocol in WBSNs, especially for applications that transmit data periodically, e.g., every 30 min or every hour. This way, each node does not have to maintain a routing table when there is no data traffic, thus, reducing the routing overhead. For a resource-limited environment, such as WBSNs, this approach needs to be considered.

Another issue to deal with is in the link model assumption. All the routing protocols proposed so far assume the link between nodes to be bi-directional, that is, each pair of nodes is able to transmit data to each other. However, in the real environment, due to node heterogeneity, the link is most likely to be uni-directional. There is no guarantee that if node A can transmit data to node B, node B can also transmit data to node A. This situation also needs to be coped with by routing protocols in WBSNs. The mechanism to transmit the data using an optimal power level of transmission is also an interesting issue to be explored.

## 5. Conclusions

In this paper, temperature-aware routing protocols in WBSNs have been reviewed. The pros and cons of each routing protocol have been analyzed. We have also discussed some perspectives on how a routing protocol in WBSNs should be designed. Then, the challenging open issues in designing a routing protocol for WBSNs were discussed.

WBSNs have the potential to improve human life quality, especially for elderly people. It is expected that in the future, using WBSNs, the health of people can be continuously monitored and ambulatory without requiring them to go to healthcare facilities. However, the implementation of sensor nodes on the human body is not as simple as it seems. Many factors must be considered, which relate closely to human health, such as tissue heating and radiation problems caused by the nodes. Nevertheless, many studies are taking place to achieve the best way possible to implement WBSNs on the human body. Studies on the design of sensor nodes, MAC protocols and routing protocols are currently under way.

We believe that a routing protocol in WBSNs must first prioritize the reduction of bioeffects as the utmost objective. The nodes are placed really close and even inside the human body. This means that human safety becomes the main concern. After the human safety concern is achieved, the other objectives, such as delay, reliability, energy efficiency or QoS, can be taken into account.

## Figures and Tables

**Table 1.** Comparisons of temperature-aware routing protocols. HPR, Hotspot Preventing Routing; TSHR, Thermal-Aware Shortest Hop Routing; RAIN, Routing Algorithm for Network of Homogeneous and ID-less Biomedical Sensor Nodes.

**Routing Protocol**	**Routing Approach**	**Application Area**	**Packet Discarding Mechanism**	**Routing Decision**	**Addressing Scheme**	**Link Model**
TARA	table-driven	*in vivo* nodes	not available	per-hop basis	global-ID	bi-directional
LTR	table-driven	*in vivo* nodes	available	per-hop basis	global-ID	bi-directional
ALTR	table-driven	*in vivo* nodes	not available	per-hop basis	global-ID	bi-directional
LTRT	table-driven	*in vivo* nodes	not available	end-to-end basis	global-ID	bi-directional
HPR	table-driven	*in vivo* nodes	available	per-hop basis	global-ID	bi-directional
TSHR	table-driven	*in vivo* nodes	not available	per-hop basis	global-ID	bi-directional
RAIN	table-driven	*in vivo* nodes	available	per-hop basis	local-ID	bi-directional
